# Aspergillus flavus endocarditis and meningitis in a child with marfan syndrome

**DOI:** 10.18502/cmm.6.4.5441

**Published:** 2020-12

**Authors:** Azam Fattahi, Shirin Sayyahfar, Ensieh Lotfali, Reza Ghasemi, Hojjat Mortezaeian

**Affiliations:** 1 Center for Research and Training in Skin Diseases and Leprosy, Tehran University of Medical Sciences, Tehran, Iran; 2 Research Center of Pediatric Infectious Diseases, Institute of Immunology and Infectious Diseases, Iran University of Medical Sciences, Tehran, Iran; 3 Department of Medical Parasitology and Mycology, School of Medicine, Shahid Beheshti University of Medical Sciences, Tehran, Iran; 4 Student Research Committee, School of Medicine, Shahid Beheshti University of Medical Sciences, Tehran, Iran; 5 Rajaie Cardiovascular, Medical, and Research Center, Iran University of Medical Sciences, Tehran, Iran

**Keywords:** *Aspergillus flavus*, Endocarditis, Fluconazole, Meningitis

## Abstract

**Background and Purpose:**

*Aspergillus* species are implicated as the etiology of approximately 26% of endocarditis cases. Central nervous system aspergillosis is a life-threatening condition that has a mortality rate of 80%.

**Case report:**

Herein, we report a four– year- old female who was admitted to the pediatric infectious ward due to a fever of unknown origin in January 2020. She was a known case of Marfan syndrome with a family history of this syndrome in her mother. The species was identified using (PCR) and the antifungal susceptibility test was performed using four antifungal agents based on the Clinical and Laboratory Standards Institute M38 3rd edition. Fluconazole-resistant *Aspergillus flavus* was identified to be responsible for endocarditis and meningitis as well as fever of unknown origin.

**Conclusion:**

The clinicians should be aware and consider fungal endocarditis in blood culture-negative endocarditis even in patients with no significant risk factor when antibiotic therapy fails.

## Introduction

Incidence of invasive fungal infections has progressively increased over the past few decades ( 1). Invasive aspergillosis (IA) is associated with the highest rate of morbidity and mortality in the immunocompromised population ( 2). Endocarditis and meningitis secondary to systemic *Aspergillus* infection constitute the severe manifestations of IA which bear a poor prognosis. Fungal endocarditis results in echogenic large bulky valvular mass and may present with acute left ventricular failure and dyspnea ( 3). 

Nearly 5% of the reported fungal endocarditis belong to the pediatric age group ( 4). *Aspergillus* species (spp.) are the etiology of approximately 21% of *Aspergillus* endocarditis cases in neonates ( 5). *Aspergillus fumigatus* is known as the most common etiologic agent (8%), followed by *Aspergillus flavus* (2%), *Aspergillus niger* (2%), and *Aspergillus terreus* (1%) ( 5). The aortic valve is the most common site of cardiac involvement (e.g., the aortic ring abscesses); however, the mitral valve (MV) involvement is rare. The survival rate depends on the underlying diseases, site of involvement, type of organism, and treatment ( 6). 

The central nervous system (CNS) aspergillosis is a life-threatening condition that has amortality rate of more than 80% ( 7).However, little is known about this serious infection in the pediatric age group ( 8).The CNS-invasive aspergillosis might be manifested with meningitis, cerebritis, abscesses, granulomas, or mycotic aneurysms ( 9). Moreover, it is mainly caused by *A. fumigatus* and *A. flavus* and rarely by *A. terreus*, *Aspergillus oryzae*, *Aspergillus granulosus*, and *Aspergillus candidus* ( 10, 11). Delayed or missed diagnosis, long duration of symptoms before hospitalization, as well as extra cardiac and CNS manifestations usually lead to death ( 2). 

Herein, the authors report the case of a four-year-old female who was admitted to the pediatric infectious ward with a fever of unknown origin in January 2020. 

## Case report

A four-year-old female patient was admitted to the pediatric infectious ward due to a fever of unknown origin in January 2020. She was a known case of Marfan syndrome with a family history of this syndrome in her mother. Both of them had a history of anterior lens dislocation that led to surgery. 

She also had a history of congenital heart disease, including atrial septal defect type 2 (ASD2), mitral regurgitation (MR), and MV prolapse regarding fever, headache, and meningeal signs a lumbar puncture was performed. She was treated for probable bacterial meningitis since the result of her cerebrospinal fluid (CSF) test was positive and active; moreover, the white blood cell was observed in CSF fluid. 

**Figure 1:**
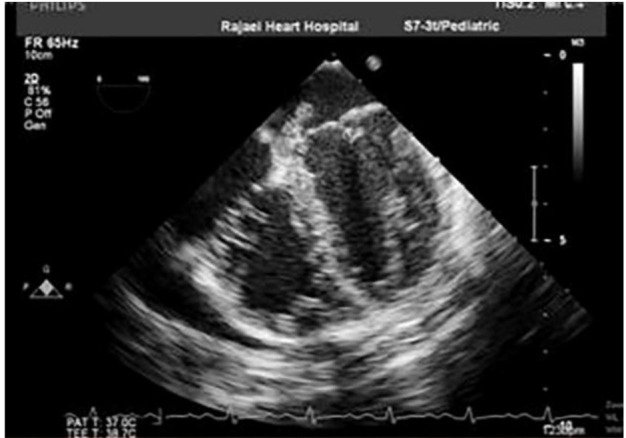
Subcostal 4 Chamber view showed large fungal vegetation connected to anterior mitral leaflet hinge point and mild pericardial effusion.

**Figure 2:**
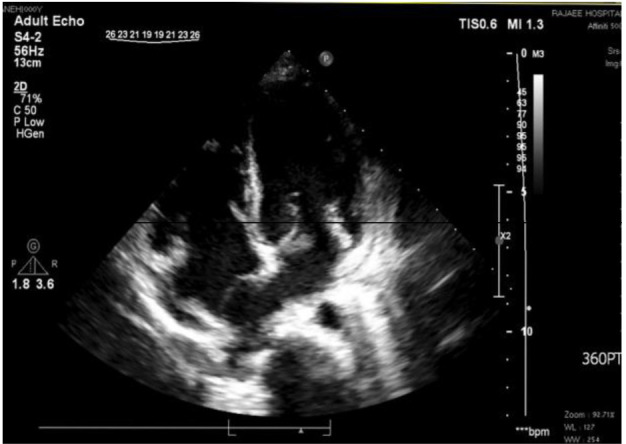
Apical 4 Chamber view showed large fungal vegetation closed to the anterior mitral leaflet hinge point

**Figure 3:**
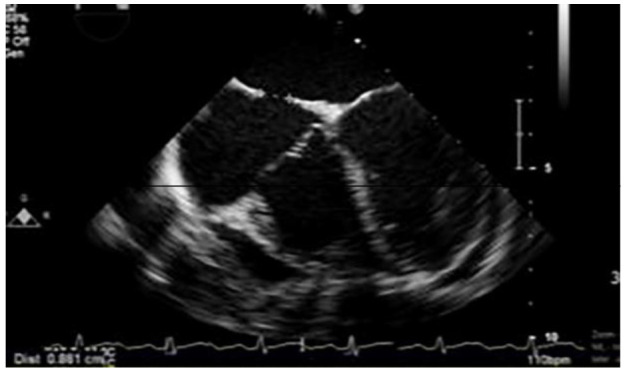
Transthoracic echocardiography in global view showed Atrial Septal Defect type 2

After five days and secondary to the changing of heart murmur intensity that revealed progressive severe MR, she was referred to a tertiary referral center for the evaluation of cardiac involvement. Transthoracic echocardiography (TTE) revealed a large dense heterogeneous and oscillating mass (18 mm×12 mm in size) attached to the atrial septum closed to the hinge point of the anterior leaflet of MV with independent movement towards the heart valve (an argument for the fungal endocardial vegetation) (Figures 1,2,3). 

Results of color Doppler flow imaging revealed an abnormal regurgitation flow at the areas of A3 anterior leaflet of mitral suggestive of perforation of MV. In addition, valvular destruction, perforation of the anterior leaflet at the site of the lesion, as well as the existence of perivalvular infection and severe eccentric MR reduced left ventricular ejection fraction. Sub-systemic pulmonary hypertension was found with mild pericardial effusion due to acute heart failure, previous history of congenital heart disease, and ASD2. 

Neurologic physical examination for the evaluation of meningeal involvement contains clinical examination and paraclinical data. The cerebrospinal fluid analysis showed meningeal irritation and brain magnetic resonance imaging revealed a small subcortical focus of involvement probably due to post-endocarditis embolic lesions. 

Infective endocarditis (IE) was diagnosed according to the DUKE criteria. Regarding the clinical and paraclinical criteria, one major criterion and two minor criteria were observed. Persistent fever and the presence of refractory heart failure due to severe acute MR led to surgery for vegetation removal, MV repair, and ASD closure. Surgical findings in the operation room indicated large fungal vegetation with perforation in the infection site. She was discharged after daily slow intravenous (IV) injection of liposomal amphotericin B dose 0/3 mg/kg for four-week. Moreover, the daily consumption of oral antifungal Voriconazole 200 mg film-coated tablets was continued for one month at home. 

The results of the present study showed in vitro resistance to fluconazole (minimum inhibitory concentration> 16 µg/mL). The TTE in the outpatient clinic after two weeks, as well as one, three, and six months after discharge showed no recurrence except mild eccentric non-significant MR. 

Tables 1 summarize the results of the hematologic indices. Biochemical test results revealed creatinine 0.3L mg/dl (NR: 0.6-1.4); AST(SGOT) 70H IU/L (NR 5-40); ALT(SGPT) 83H IU/L (NR 5-40).

The peripheral blood sample was taken and inoculated into an anaerobic blood culture medium (manufactured in BC, USA) at 30ºC for five days in an automated blood culture system (manufactured in BC, USA); at 30ºC for five days; however, no growth was observed. The resected vegetation was inoculated into formalin and normal saline solution. The pathology result revealed a fibrohyalinized tissue with an area of fibrinoleukocytic exudate, mixed inflammatory cell infiltration, and neovascularization consistent with endocarditis. Furthermore, the hematoxylin and eosin staining showed a few thin hyphae-like elements. To confirm the fungal infection, the second portion of the specimen was sent to the Medical Mycology Laboratory of the Center for Research and Training in Skin Diseases and Leprosy in Tehran, Iran for further evaluations. 

**Table 1:** Hematology information

General Hematology		
Test	Unit	Result
Complete CBC		
Hemoglobin	9.8	g/dl
Hematocrit	30.7	
Red Blood Cell	4.21	10^6 cells/mm^3^
White Blood Cell	26300	cells/mm^3^
Diff		
Platelet count	625	10^3/mm^3^
Red Blood Cell Index		
Mean corpuscular hemoglobin concentration	31.9	g/dl
Mean Corpuscular Volume	72.9	Fl
Mean Corpuscular Hemoglobin	23.3	Pg
Erythrocyte sedimentation rate	45	mm/h

Initially, the biopsy specimen of the MV was taken and a sub-culture was made on Sabouraud Dextrose Agar (SDA) (manufactured in Merck, Germany) at 35ºCfor seven days. After seven days, the colonies of *Aspergillus* spp. were grown in the SDA medium. The PCR for the amplification of the β-tubulin gene was conducted to identify the species precisely. Genomic DNA was obtained from the colonies cultured on the Czapek’s Agar (manufactured in Merck, Germany) forseven days at 35ºC using the genomic DNA extraction kit (manufactured in Roche Life Science, Germany). 

In this study, the amplification of the β-tubulin gene was performed using the β-tubulin forward and β-tubulin reverse primers ( 12). The PCR amplicon was subjected to sequence using the same primers. Subsequently, the sequences were compared with reference data available from the Gene Bank database using the BLAST sequence search tool (http://www.ncbi.nlm.nih.gov/BLAST). 

The antifungal susceptibility test was performed by the broth microdilution methodusing four antifungal agents, including voriconazole, itraconazole, fluconazole, and amphotericin B (manufactured in Sigma-Aldrich, USA). Based on the Clinical and Laboratory Standards Institute (CLSI) M38 3rd ed ( 13), *Candida parapsilosis* (ATCC 22019) was chosen as a quality control strain in every run. 

The culture on SDA revealed green and powdery surface colonies. The microscopic examination of the colonies was compatible with *A.flavus*. The sequencing result was interpreted and deposited in the GenBank under accession no: MW195498. 

The *A. flavus* strain showed resistance to fluconazole with minimum inhibitory concentration (MIC>16 µg/mL). Furthermore, susceptibility to voriconazole (MIC 0.313 µg/mL), amphotericin B (MIC 0.313 µg/ML), and itraconazole (MIC 0.25 µg/mL) was noted in this study.

The patient was first treated with vancomycin and ceftriaxone which was later switched to the combination of meropenem, ciprofloxacin, and vancomycin when the fever continued and the clinical status deteriorated. The patient was treated with liposomal amphotericin B regarding the result of cultures and the diagnosis of IA. She was discharged after four weeks of treatment with IV amphotericin B. It is worth mentioning that the antifungal treatment was continued with oral voriconazole for an additional four weeks after the performance of the susceptibility test. 

This case report was performed in compliance with the Declaration of Helsinki and written informed consent was obtained from the legal guardians of the patient. The details will be included in the manuscript for publication. 

## Discussion

The IA typically advances subsequent to long and severe neutropenia which commonly affects the lungs and rarely influences paranasal sinuses, CNS, skin, and soft tissues. Medical signs may be nonspecific (i.e., long-lasting fever in acute neutropenia not treated with spectrum antibiotic therapy). Despite the significance of this infection, there are limited data on the epidemiology, value of diagnostic tests, and therapeutic approaches in the pediatric population when the heart and CNS are involved. 

Early and accurate diagnosis of IA affects the therapeutic strategies, which are mainly derived from clinical studies on adults. When fungal meningitis is suspected, the choice of the antifungal drug should be restricted to agents that sufficiently pass the blood-brain-barrier, such as the lipid formulations of amphotericin B (e.g., amphotericin B lipid complex and liposomal amphotericin B) or the broad-spectrum triazole voriconazole ( 14, 15). 

Given the results of a randomized controlled trial on adults, voriconazole is a superior choice for the treatment of IA, including cardiac and CNS infections ( 16). No pediatric randomized trial has assessed the outcome of cardiac and CNS infections treated with voriconazole. Some studies have reported the successful outcome of IA management with voriconazole in the pediatric age group ( 17). Our patient was successfully treated with combination therapy (i.e., surgery and IV liposomal amphotericin B followed by four weeks of oral voriconazole). Results of the present study indicated*in vitro*resistance to fluconazole (MIC>16 µg/mL). The cases of *A. flavus* endocarditis among children and the outcome of treatment are summarized in Table 2 . 

**Table 2:** *Aspergillus flavus* endocarditis cases reported among children: CLINICAL aspects

NO.	Source	Age/ gender	Comorbidity	Involving site	Diagnostic modality	Initial presentation	Clinical findings	Surgical treatment	Antifungal treatment	Duration of treatment	Outcome
1	Kamer et al.^18^	13 Y.O./F	Mitral valve replacement	Mitral valve	Histology and culture of embolus	Fever, right hemiparesis, aphasia, subconjunctival hemorrhage,	Polymorphonuclear leukocytosis, anemia, pyuria, microscopic hematuria, emboli to major vessels, especially to the central nervous system and extremities	Yes	AMB	10 days (continued until death)	Died
2	Kennedy et al. 19	6 Y.O./M	Stage 4 neuroblastoma	Right ventricle with the involvement of the chordae	Blood culture	Cough, weight loss, anorexia, lethargy, pain in the hip, difficulty in walking, septic skin spots, pyrexia	Anemia, low platelets, leucoerythroblastic blood film, increased urinary catecholamines	No	AMB, ITR	14 weeks	Died due to progressive malignancy disease 8 months after diagnosis of endocarditis
3	Rao et al. 20	11 Y.O./M	Refractory AML	Right ventricle	Histology and culture of debrided tissue	Pyrexia, necrotic lesion on nose, fever	Vegetation on the right ventricular wall, generalized opacity in CXR, bilateral hazy infiltrates in CT	No	AMB, ITR	N.M.	Survived
4	Navabi et al. 21	2.5 Y.O./M	Tetralogy of Fallot	Main pulmonary artery	Histology	Hypotension, fever	Hepatomegaly, renal failure, liver failure, leukocytosis, bandemia, thrombocytopenia, bilateral pulmonary expansion, cardiac arrest	No	Died prior to initiation of treatment	-	Died
		10 M.O./F	Tetralogy of Fallot	Vegetation on VSD patch	Histology and culture of debrided tissue	Chills and fever,	Increased CRP level,	No	AMB	9 weeks	Died after 13 weeks

Y.O.: year old, M.O.: month old, F: female, M: male, AMB: Amphotericin B, ITR: itraconazole, AML: acute myeloid leukemia, N.M.: not mentioned, CXR: chest X-Ray, CT: computerized tomography,VSD: ventricular septal defect

## Conclusion

According to the results of this case report, the optimization of both diagnosis and treatment leads to a better outcome of IA in children. Based on the findings of this study and the aforementioned studies worldwide, after obtaining approved antifungal test results, fluconazole can be regarded as a suitable first-line choice for the treatment of IA. 

## Author’s contribution:

 H. M. and Sh. S. obtained the sample from patient and interpreted it. A. F. and EL. R. GH. collected the data and drafted of the manuscript. A. F., H. M., and SH. S. critically revised the manuscript. A. F. processed the laboratory examination.

##  Financial disclosure:

This study was not financially supported.

## References

[1] Meshaal MS, Labib D, Said K, Hosny M, Hassan M, Al Aziz  SA, et al ( 2018). Aspergillus endocarditis: diagnostic criteria and predictors of outcome, a retrospective cohort study. PloS One.

[2] Ghajari  A, Lotfali E, Azari M, Fateh R, Kalantary S ( 2015). Fungal airborne contamination as a serious threat for respiratory infection in the hematology ward. Tanaffos.

[3] Abuzaid AA, Zaki  M, Tarif H ( 2015). Atypical early Aspergillus endocarditis post prosthetic mitral valve repair: a case report. Heart Views.

[4] Soler JAV, Camacho WJM, Rodríguez CXF, Mejía  JAN, Guerrero CF, Camacho MAM ( 2018). Aspergillus flavus endocarditis in an immunocompetent child. Case report. MedMycolCase Rep.

[5] Millar BC, Jugo J, Moore JE  ( 2005). Fungal endocarditis in neonates and children. Pediatr Cardiol.

[6] Ellis M, Al-Abdely H, Moore  JE ( 2001). Fungal endocarditis: evidence in the world literature, 1965–1995. Clin Infect Dis.

[7] Dotis J, Iosifidis E, Roilides E ( 2007). Central nervous system aspergillosis in children: a systematic review of reported cases. Int J Infect Dis.

[8] Denning D W ( 1998). Invasive aspergillosis. Clin Infect Dis.

[9] Shuper A, Levisky HI, Cornblath DR ( 1991). Early invasive CNS aspergillosis. An easily missed diagnosis. Neuroradiology.

[10] Antinori S, Corbellino M, Meroni L, Resta F, Sollima S, Tonolini M, et al (2013). Aspergillus meningitis: a rare clinical manifestation of central nervous system aspergillosis. Case report and review of 92 cases. J Infect.

[11] Elsawy  A, Faidah H, Ahmed A, Mostafa A, Mohamed F ( 2015). Aspergillus terreus meningitis in immunocompetent patient: a case report. Front Microbiol.

[12] Nasri T, Hedayati MT, Abastabar M, Pasqualotto AC, Armaki MT, Hoseinnejad A, et al ( 2015). PCR-RFLP on β-tubulin gene for rapid identification of the most clinically important species of Aspergillus. J Microbiol Methods.

[13] Clinical and Laboratory Standards Institute (2017). Reference method for broth dilution antifungal susceptibility testing of Filamentous Fungi; approved standard. CLSI document M38.

[14] Walsh TJ, Lutsar I, Driscoll T, Dupont B, Roden M, Ghahramani P, et al ( 2002). Voriconazole in the treatment of aspergillosis, scedosporiosis and other invasive fungal infections in children. Pediatr Infect Dis J.

[15] Haßler A, Porto L, Lehrnbecher T ( 2015). Cerebral fungal infection in pediatric cancer patients. Curr Fungal Infect Rep.

[16] Herbrecht R, Denning DW, Patterson TF, Bennett JE, Greene RE, Oestmann JW, et al ( 2002). Voriconazole versus amphotericin B for primary therapy of invasive aspergillosis. N Eng J Med.

[17] Yenişehirli G, Bulut Y, Güven M, Günday E ( 2009). In vitro activities of fluconazole, itraconazole and voriconazole against otomycotic fungal pathogens. J Laryngol Otol.

[18] Kammer  RB, Utz JP ( 1974). Aspergillus species endocarditis: the new face of a not so rare disease. AmJ Med.

[19] Kennedy HF, Simpson EM, Wilson N, Richardson MD, Michie JR ( 1998). Aspergillus flavus endocarditis in a child with neuroblastoma. J Infect.

[20] Rao  K, Saha V ( 2000). Medical management of Aspergillus flavus endocarditis. Pediatr Hematol Oncol.

[21] Navabi  MA, Ajami H, Amirghofran  A, Peyravian F ( 1998). Aspergillus endocarditis: rare but serious Aspergillus ball obstructing the pulmonary artery. EurJ Cardiothorac Surg.

